# Heterogeneous echogenicity of the underlying thyroid parenchyma: how does this affect the analysis of a thyroid nodule?

**DOI:** 10.1186/1471-2407-13-550

**Published:** 2013-11-16

**Authors:** Mina Park, So Hee Park, Eun-Kyung Kim, Jung Hyun Yoon, Hee Jung Moon, Hye Sun Lee, Jin Young Kwak

**Affiliations:** 1 Department of Radiology, Research Institute of Radiological Science, Yonsei University, College of Medicine, 50 Yonsei-ro, Seodaemun-gu, Seoul 120-752, South Korea; 2 Biostatistics Collaboration Unit, Medical Research Center, Yonsei University College of Medicine, Seoul 120-752, South Korea

**Keywords:** Ultrasonography, Thyroid gland, Diffuse thyroid disease, Thyroid malignancy, Thyroid nodule

## Abstract

**Background:**

Heterogeneous echogenicity of the thyroid gland has been associated with diffuse thyroid disease and benign and malignant nodules can coexist with diffuse thyroid disease. Underlying heterogeneous echogenicity might make it difficult to differentiate between benign and malignant nodules on US. Thus, the aim of this study was to evaluate the influence of underlying thyroid echogenicity on diagnosis of thyroid malignancies using US.

**Methods:**

A total of 1,373 patients who underwent US-guided fine needle aspiration of 1,449 thyroid nodules from June 2009 to August 2009 were included. The diagnostic performance of US assessment for thyroid nodules was calculated and compared according to underlying thyroid echogenicity. The diagnostic performance of US assessments in the diagnosis of thyroid malignancy according to the underlying parenchymal echogenicity was compared using a logistic regression with the GEE (generalized estimating equation) method. Each US feature of malignant and benign thyroid nodules was analyzed according to underlying echogenicity to evaluate which feature affected the final diagnosis.

**Results:**

Among the 1,449 nodules, 325 (22.4%) were malignant and 1,124 (77.6%) were benign. Thyroid glands with heterogeneous echogenicity showed significantly lower specificity, PPV, and accuracy compared to thyroid glands with homogeneous echogenicity, 76.3% to 83.7%, 48.7% to 60.9%, and 77.6% to 84.4%, respectively (*P* = 0.009, 0.02 and 0.005, respectively). In benign thyroid nodules, microlobulated or irregular margins were more frequently seen in thyroid glands with heterogeneous echogenicity than in those with homogenous echogenicity (*P* < 0.001).

**Conclusion:**

Heterogeneous echogenicity of the thyroid gland significantly lowers the specificity, PPV, and accuracy of US in the differentiation of thyroid nodules. Therefore, caution is required during evaluation of thyroid nodules detected in thyroid parenchyma showing heterogeneous echogenicity.

## Background

Heterogeneous echogenicity of the thyroid gland has been associated with diffuse thyroid disease (DTD) including Hashimoto thyroiditis (HT) and Graves’ disease [1-4]. Ultrasonographic (US) features of HT have been reported to show a broad spectrum of abnormal features ranging from focal ill-defined hypoechoic areas to diffuse homogeneous hypoechoic regions showing areas of internal echogenic fibrous septa or diffuse heterogeneous hypoechogenicity showing micronodular patterns [1-4].

Benign and malignant nodules can coexist with DTD [5,6]. In particular, the association between HT and papillary thyroid carcinoma (PTC) has been reported in many studies [5,7-9]. Although US features of malignant thyroid nodules with diffuse HT have been reported to be similar to typical malignant US features [10], underlying heterogeneous echogenicity might make it difficult to differentiate between benign and malignant nodules. Besides these considerations, there are no published reports on this topic: Does underlying thyroid parenchyma echogenicity affect the analysis of a thyroid nodule? If it does, what are the associated US features impacting the analysis of a thyroid nodule?

This study investigated the influence of underlying thyroid echogenicity on the diagnosis of thyroid malignancies.

## Methods

This retrospective study was approved by the institutional review board (IRB) and ethics committee of Severance hospital, Seoul, Korea. Neither patient approval nor informed consent was required for review of medical records or images. Informed consent was signed and obtained from all patients before US-FNA or surgery prior to procedures as a daily practice.

**Figure 1:**
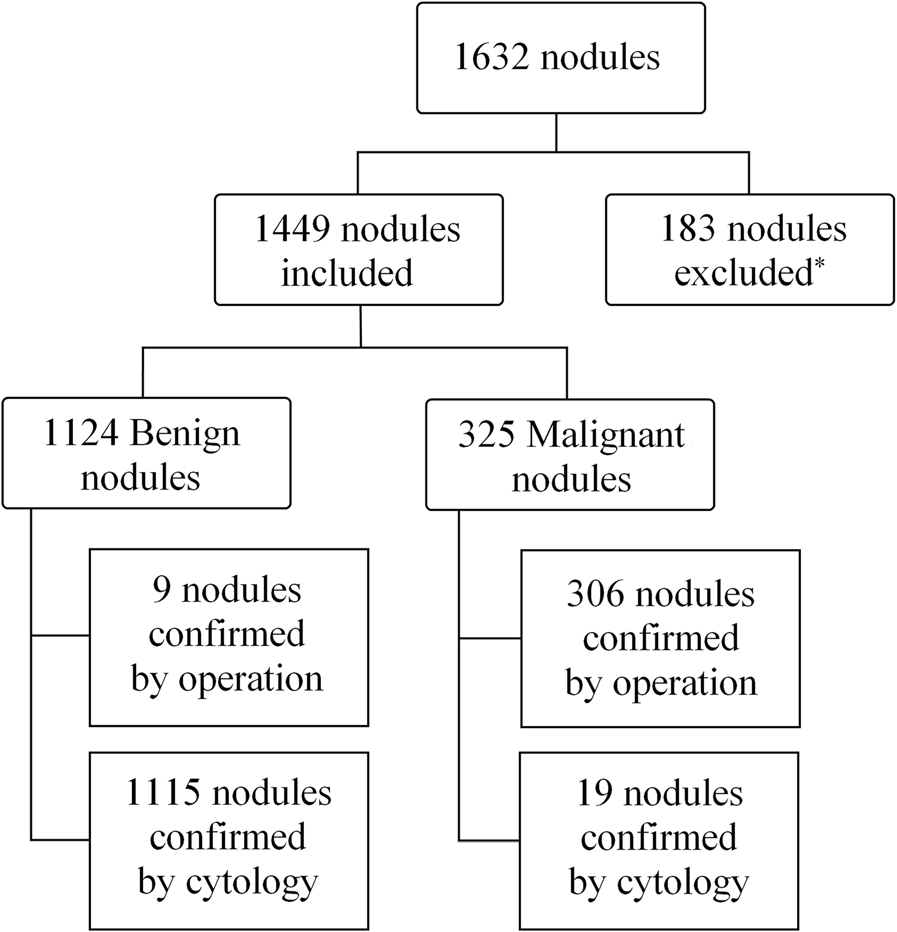
**Diagram of the study group.** *Exclusion criteria in the result.

Between June 2009 and August 2009, there were 1,534 consecutive patients with 1,632 thyroid nodules who underwent US-guided fine needle aspiration (US-FNA) on focal thyroid nodules larger than 5 mm in our institution (a referral center) in Korea. Among them, we retrospectively enrolled 1,373 patients with 1,449 thyroid nodules, from whom we could obtain cytopathologic results and follow-up data (Figure 1). There were 3 patients who underwent US-FNAs at 3 nodules, 70 patients who underwent US-FNAs at 2 nodules, and 1300 patients who underwent US-FNAs at 1 nodule. The mean age of patients included was 50.8 years (range, 15–95 years). Among the 1,373 patients, 1,126 were women (mean age, 50.5 years, range, 15–95 years) and 247 were men (mean age, 52.1 years, range, 25–80 years).

### US and US-FNA

**Figure 2:**
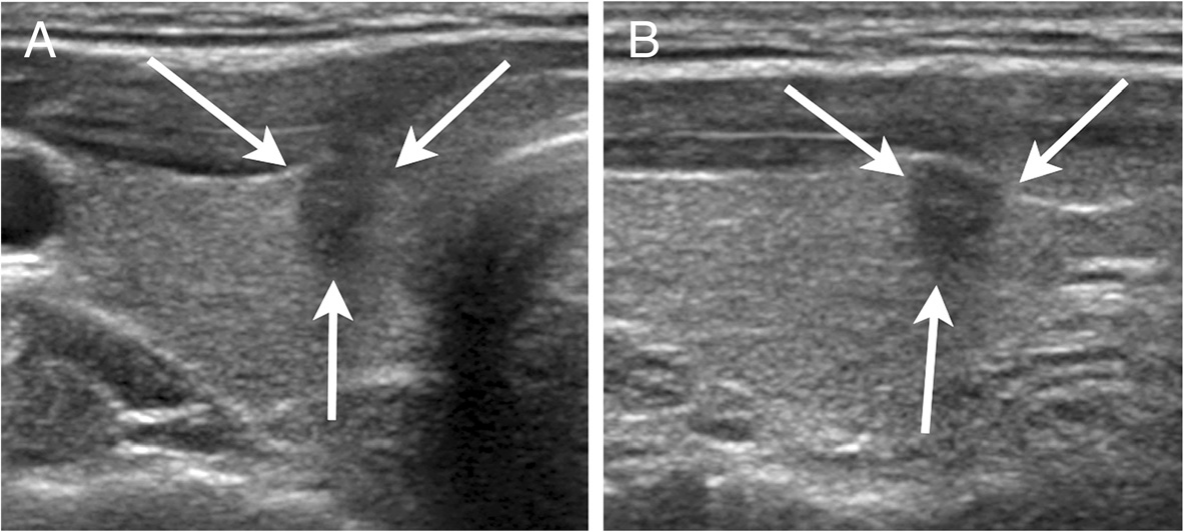
**US findings of a malignant thyroid nodule in underlying homogenous thyroid echogenicity. (a)** Transverse and **(b)** longitudinal US showed a 6-mm irregular, taller than wide nodule (arrows) with homogenous echogenicity of the underlying thyroid gland in the right thyroid gland. The lesion was diagnosed as papillary microcarcinoma on surgical histopathology.

US examinations and US-FNA were performed by one of seven board-certified radiologists with 1 to 15 years of experience in thyroid imaging, using a 7- to 15- MHz linear probe (HDI 5000, Philips-Advanced Technology Laboratories, Bothell, WA, USA) or a 5- to 12- MHz linear probe (iU22, Philips-Advanced Technology Laboratories, Bothell, WA, USA). Compound imaging was performed for all US examinations. US features of the underlying thyroid parenchyma and thyroid nodule targeted for US-FNA were assessed at the time of US examination and US-FNA. Diffuse echogenicity of the thyroid parenchyma showing numerous micronodular appearances or echogenic septations was defined as ‘heterogeneous echogenicity’ of the thyroid gland [6,11,12]. Thyroid nodules were classified according to internal component, echogenicity, margin, calcification, and shape on US. Marked hypoechogenicity, microlobulated or irregular margins, microcalcifications, and taller than wide shape were considered suspicious malignant features of thyroid nodules on US (Figure 2) [13]. When thyroid nodules had one or more of the previously mentioned suspicious malignant US features, they were classified as “positive US”. When the thyroid nodules showed no suspicious malignant features, they were classified as “negative US”. After US, each US feature was recorded by the radiologists who performed the US on provided result sheets including the underlying echogenicity of the thyroid gland on US.

At our institution, we do not routinely undergo FNA at thyroid nodules less than 5 mm. The US-FNAs were performed either on the thyroid nodule with suspicious US features or on the largest thyroid nodule if no suspicious US features were detected. However, FNAs were sometimes performed on multiple nodules in one patient because of multiple suspicious US features, physician’s or patient’s request. US-FNAs were performed using a freehand biopsy technique with a 23-gauge needle attached to a 2-ml disposable plastic syringe. Each lesion was aspirated at least twice. Aspirated material was expelled onto glass slides that were immediately placed in 95% alcohol for Papanicolaou staining. The remaining aspirated material in the syringe was rinsed with saline and processed for cell block preparation. Five experienced cytopathologists interpreted the cytology slides. In the study period, cytological reports were classified as (a) benign, (b) indeterminate, (c) suspicious for papillary thyroid carcinoma, (d) malignant, or (e) nondiagnostic. Among cases with benign cytology, lymphocytic thyroiditis was further diagnosed when the cytological specimen met the following criteria: the specimen showed grouped, monolayer sheets or scattered follicular and Hurthle cells with scattered lymphocytes; the colloid was scanty; and the follicular cells showed nuclear atypia with nuclear enlargement and clearing in the absence of nuclear grooves or inclusions [14]. A non-diagnostic cytology result was defined as the presence of less than six groups of cells, each containing at least ten cells [15,16]. Indeterminate cytology included follicular or Hurthle cell neoplasm. The “suspicious for papillary carcinoma” cytological result was designated when the specimen exhibited cytological atypia (nuclei are crowded and overlapping, enlarged, and pleomorphic) but showed insufficient cellularity for definite diagnosis of papillary carcinoma [17]. For this study, we recorded the results by retrospectively reviewing the cytological reports.

### Measurement of serum anti-thyroid autoantibodies

Anti-thyroid antibodies were evaluated using venous blood samples from 938 patients. Serum thyroid peroxidase antibody (TPOAb), thyroglobulin antibody (TgAb) and TSH-binding inhibitory immunoglobulins (TBII) levels were measured by radioimmunoassay (Brahms, Hennigsdorf/Berlin, Germany). The existence of TPOAb and/or TgAb was defined by a serum concentration of the relevant thyroid autoantibody > 60 IU/L. Patients with HT were defined by positive results for TPOAb and/or TgAb [18]. A TBII exceeding 10% was considered positive. Patients with Graves’ disease were defined as positive for TBII.

### Statistical analysis

Histopathology results from surgery or US-FNA cytology were considered the standard reference of thyroid nodules. Statistical comparisons were performed using the Chi-square test for categorical variables and independent *t*-test for continuous variables. The diagnostic performance of US assessments of thyroid nodules according to the echogenicity of underlying thyroid parenchyma was calculated, including sensitivity, specificity, positive predictive value (PPV), negative predictive value (NPV), and accuracy. The diagnostic performance of US assessments in the diagnosis of thyroid malignancy according to the underlying parenchymal echogenicity was compared using a logistic regression with the GEE (generalized estimating equation) method. We considered *P*-values less than 0.05 statistically significant. Statistical analysis was performed using commercial statistical software (SAS version 9.1, SAS Inc., Cary, NC, USA).

## Results

We retrospectively enrolled 1,632 thyroid nodules from 1,534 patients, from whom we could obtain cytologic results. We excluded 125 nodules with non-diagnostic results of FNA, 16 nodules with atypical follicular epithelial cells, 2 nodules with results of parathyroid cells and lymph nodes, and 40 nodules without US findings available. Among the 1,449 nodules, 325 (22.4%) were malignant and 1,124 (77.6%) were benign (Figure 1). Histopathologic diagnoses of the 315 thyroid nodules are listed in Table 1. Patients (51.6 ± 11.8 years) diagnosed with benign nodules were significantly older than those (47.8 ± 12.6 years) diagnosed with malignant nodules (*P* < .001). The mean size of the benign nodules were 16.6 ± 10.5 mm, which was significantly larger than that of the malignant nodules, 11.8 ± 8.6 mm (*P* < .001). Gender was not associated with malignancy (*P* = 0.954).

**Table 1:** Histopathologic diagnosis of 315 thyroid nodules

**Final pathology**	**n (%)**
**Malignant (n = 306, 97.1%)**	
Papillary carcinoma, conventional	264 (86.3)
Papillary carcinoma, follicular variant	29 (9.5)
Papillary carcinoma, diffuse sclerosing variant	5 (1.6)
Papillary carcinoma, oncocytic variant	3 (1.0)
Medullary carcinoma	3 (1.0)
Follicular carcinoma	1 (0.3)
Hűrthle cell carcinoma	1 (0.3)
**Benign (n = 9, 2.9%)**	
Adenomatous hyperplasia	8 (88.9)
Hyalinizing trabecular adenoma	1 (11.1)

The mean age (52.3 ± 12.4 years) of the patients with underlying heterogeneous echogenicity of the thyroid gland was older than that (50.4 ± 12 years) of the patients with underlying homogeneous thyroid echogenicity (*P* = 0.015). In the underlying heterogeneous echogenicity group, 270 were women and 28 were men while in the underlying homogenous echogenicity group, 856 were female and 219 were male, exhibiting female predominancy in the underlying heterogeneous echogenicity group (*P* < .001). The mean size (15.6 ± 10.5 mm) of the nodules in the underlying homogenous echogenicity group was larger than that (14.7 ± 9.2 mm) of underlying heterogeneous echogenicity group, but it was not statistically significant (*P* = 0.119).

**Figure 3:**
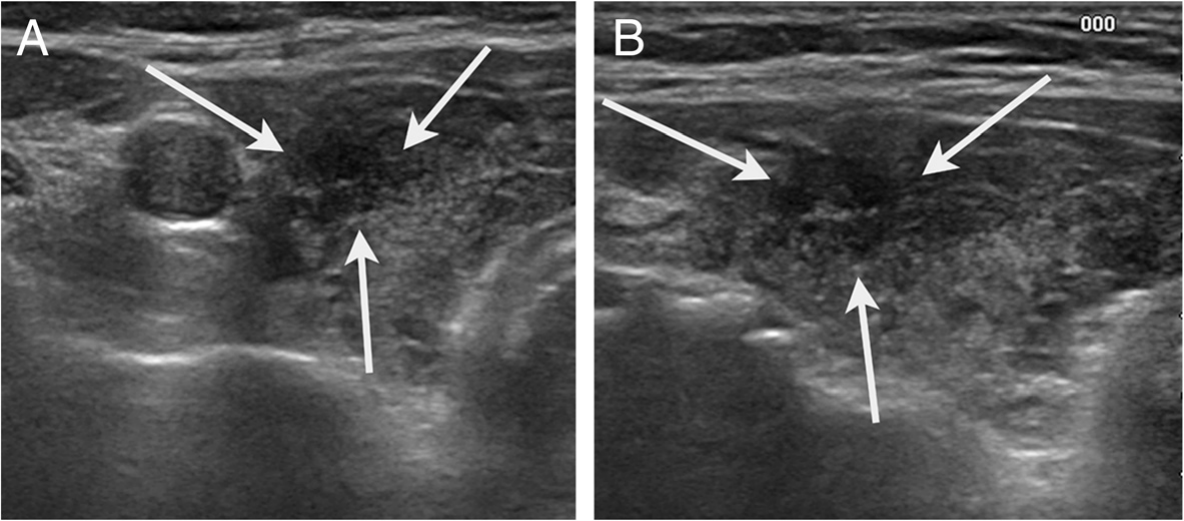
**US finding of a false positive case with underlying heterogeneous thyroid gland. (a)** Transverse and **(b)** longitudinal US showed 7-mm irregular, hypoechoic nodule (arrows) in heterogeneous echogenicity of the underlying thyroid gland in the left thyroid gland. The lesions was diagnosed with adenomatous hyperplasia on fine-needle aspiration biopsy and showed decrease in size on 5 years follow-up US.

**Figure 4:**
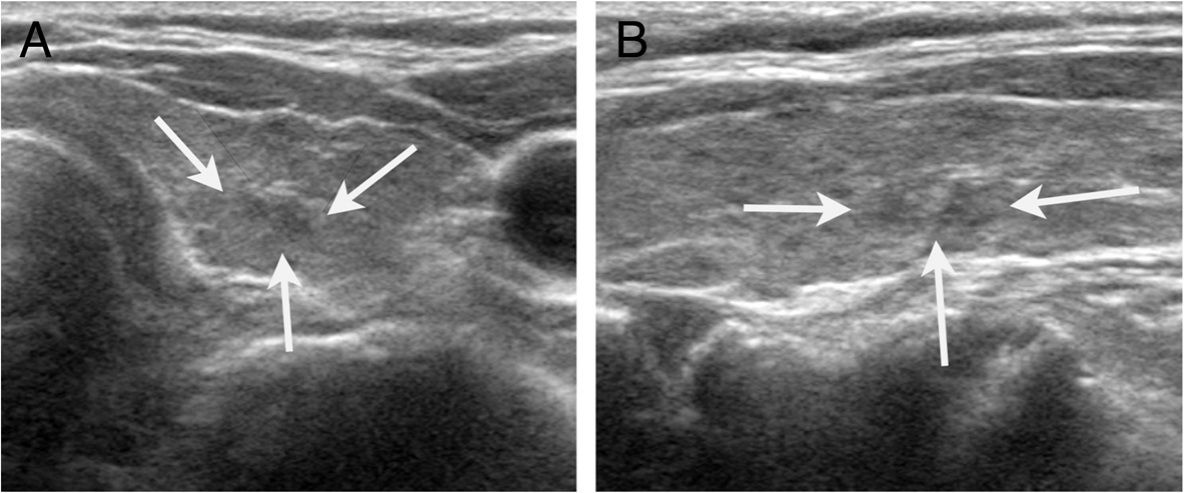
**US finding of a false positive case with underlying heterogenous thyroid gland echogenicity. (a)** Transverse and **(b)** longitudinal US showed an 8-mm microlobulated, marked hypoechoic nodule (arrows) with heterogenous echogenicity of the underlying thyroid gland. This lesion was later found to be lymphocytic thyroditis on fine-needle aspiration biopsy and no longer detectable on 2 years follow up US.

Of the 1,449 nodules included, 317 (21.9%) showed underlying heterogeneous echogenicity of the thyroid parenchyma on US. Table 2 shows the diagnostic performance of US in the differential diagnosis of thyroid nodules, comparing the two groups with and without underlying heterogeneous echogenicity of the thyroid parenchyma on US. The thyroid nodules in a thyroid gland with heterogeneous echogenicity had a significantly lower specificity, PPV and accuracy compared to those with homogeneous echogenicity. There were no significant differences in sensitivity and NPV between the two groups. To document the reason for different diagnostic performances of US according to the echogenicity of the thyroid parenchyma, we analyzed each US feature by the malignant and benign thyroid group according to the underlying echogenicity of the thyroid gland (Table 3). In benign thyroid nodules, microlobulated or irregular margins on US were more frequently seen in nodules in a thyroid gland with heterogeneous echogenicity than in those with homogenous echogenicity (*P* < 0.001) (Figure 3). On the other hand, in malignant thyroid nodules, there were no significant differences in US features according to the underlying echogenicity of the thyroid gland. Among a total of 1124 nodules diagnosed as benign thyroid nodules, 875 nodules were seen in the background of homogenous thyroid echogenicity and the other 249 nodules were found in that of heterogeneous thyroid echogenicity. The nodules that were diagnosed as lymphocytic thyroiditis occurred in 1.5% (13/875) of underlying homogenous thyroid echogenicity, while under heterogeneous thyroid echogenicity 14.9% (37/249) were found to be focal lymphocytic thyroiditis (Figure 4). The margins of nodules with lymphocytic thyroiditis were microlobulated or irregular in 8 nodules with underlying homogeneous thyroid echogenicity and 16 nodules with underlying heterogeneous thyroid echogenicity. We also analyzed each US feature of 1399 nodules according to underlying thyroid gland echogenicity while excluding nodules which were diagnosed with lymphocytic thyroiditis to eliminate the lymphocytic thyroiditis effect on US diagnostic performance (Table 4). In benign thyroid nodules, we could still more often find microlobulated or irregular margin on US in nodules with heterogeneous thyroid echogenicity than in those with homogenous thyroid echogenicity (*P* = 0.007).

**Table 2:** Diagnostic performance of US assessment in thyroid nodules according to the underlying echogenicity of the thyroid parenchyma

	**Heterogeneous echogenicity on US**	**Homogeneous echogenicity on US**	***P*-value**
Sensitivity	82.4% (56/68)	86.8% (223/257)	0.354
Specificity	76.3% (190/249)	83.7% (732/875)	0.009
Positive predictive value	48.7% (56/115)	60.9% (223/366)	0.02
Negative predictive value	94.1% (190/202)	95.6% (732/766)	0.378
Accuracy	77.6% (246/317)	84.4% (955/1132)	0.005

**Table 3:** Comparison of each US feature of 1449 thyroid nodules according to underlying echogenicity

	**Total**			**Malignant**			**Benign**		
	**Heterogeneous echogenicity**	**Homogeneous echogenicity**	***P*-value**	**Heterogeneous echogenicity**	**Homogeneous echogenicity**	***P*-value**	**Heterogeneous echogenicity**	**Homogeneous echogenicity**	***P*-value**
Total number	317	1132		68	257		249	875	
Echogenicity			0.078			0.174			0.068
Hyper/isoechogenicity	125 (39.4)	525 (46.4)		3 (4.4)	28 (10.9)		122 (49.0)	497 (56.8)	
Hypoechogenicity	176 (55.5)	549 (48.5)		56 (82.4)	186 (72.4)		120 (48.2)	363 (41.5)	
Marked hypoechogenicity	16 (5.1)	58 (5.1)		9 (13.2)	43 (16.7)		7 (2.8)	15 (1.7)	
Margin			0.005			0.314			<0.001
Well circumscribed	203 (64.0)	818 (72.3)		12 (17.7)	60 (23.4)		191 (76.7)	758 (86.6)	
microlobulated or irregular	114 (36.0)	314 (27.7)		56 (82.4)	197 (76.7)		58 (23.3)	117 (13.4)	
Calcifications			0.849			0.986			0.555
Microcalcifications	42 (13.3)	141 (12.5)		29 (42.7)	108 (42.0)		13 (5.2)	33 (3.8)	
Macrocalcifications	39 (12.3)	151 (13.3)		9 (13.2)	36 (14.0)		30 (12.1)	115 (13.1)	
No calcifications	236 (74.5)	840 (74.2)		30 (44.1)	113 (44.0)		206 (82.7)	727 (83.1)	
Shape			0.737			0.790			0.245
Wider than tall	251 (79.2)	906 (80.0)		29 (42.7)	105 (40.9)		222 (89.2)	801 (91.5)	
Taller than wide	66 (20.8)	226 (20.0)		39 (57.4)	152 (59.1)		27 (10.8)	74 (8.5)	

**Table 4:** Comparison of each US feature of 1399 thyroid nodules excluding thyroid nodules with cytologic results of lymphocytic thyroiditis according to underlying echogenicity

	**Total**			**Malignant**			**Benign**		
	**Heterogeneous echogenicity**	**Homogeneous echogenicity**	***P*-value**	**Heterogeneous echogenicity**	**Homogeneous echogenicity**	***P*-value**	**Heterogeneous echogenicity**	**Homogeneous echogenicity**	***P*-value**
Total number	280	1119		68	257		212	862	
Echogenicity			0.209			0.174			0.366
Hyper/isoechogenicity	114 (40.7)	520 (46.5)		3 (4.4)	28 (10.9)		111 (52.4)	492 (57.1)	
Hypoechogenicity	152 (54.3)	543 (48.5)		56 (82.4)	186 (72.4)		96 (45.3)	357 (41.4)	
Marked hypoechogenicity	14 (5.0)	56 (5.0)		9 (13.2)	43 (16.7)		5 (2.4)	13 (1.5)	
Margin			0.012			0.314			0.007
Well circumscribed	182 (65.0)	813 (72.7)		12 (17.6)	60 (23.3)		170 (80.2)	753 (87.4)	
microlobulated or irregular	98 (35.0)	306 (27.3)		56 (82.4)	197 (76.7)		42 (19.8)	109 (12.6)	
Calcifications			0.728			0.986			0.613
Microcalcifications	40 (14.3)	140 (12.5)		29 (42.7)	108 (42.0)		11 (5.2)	32 (3.7)	
Macrocalcifications	37 (13.2)	149 (13.3)		9 (13.2)	36 (14.0)		28 (13.2)	113 (13.1)	
No calcifications	203 (72.5)	830 (74.2)		30 (44.1)	113 (44.0)		173 (81.6)	717 (83.2)	
Shape			0.306			0.790			0.124
Wider than tall	217 (77.5)	898 (80.3)		29 (42.6)	105 (40.9)		188 (88.7)	793 (92.0)	
Taller than wide	63 (22.5)	221 (19.7)		39 (57.4)	152 (59.1)		24 (11.3)	69 (8.0)	

The diagnosis of DTD was based on either histopathologic reports (n = 51) or serum antibody testing (n = 369). Three hundred and sixty nine patients underwent serum TPOAb and TBII tests at least three months prior to US-FNA. Patients with DTD showed significantly more heterogeneous echogenicity (39.8%) of the thyroid parenchyma on US compared with patients without DTD (13.7%, *P* < 0.001).

## Discussion

DTD encompasses diverse clinical entities including Graves’ disease and HT and it is commonly observed throughout the population. Annually around 0.5 per 1000 women develop Graves’ disease and a further 1-2% have autoimmune hypothyroidism including HT [19,20]. These disorders are 5 – 10 times more frequent in females [21] and our study also exhibits female predominancy (F:M = 4.7 :1). Both disorders share a cognate etiology with susceptibility determined by genetic factors and environmental factors but present with different clinical symptoms [21]. The most common cause of hypothyroidism is environmental iodine deficiency [22]. In areas of iodine sufficiency such as the United States and Korea, HT is the most common cause of hypothyroidism [23]. On the other hand, in European countries the atrophic variant of HT is much more common and mostly leads to hypothyroidism slowly [24]. Graves’ disease manifests as any form of hyperthyroidism with specific symptoms of Graves’ disease such as ophthalmopathy [20].

On US, a change in the underlying thyroid echotexture involving diffuse thyroid glands can help guide the diagnosis of DTD [1,25,26]. Characteristic US features of HT consist of numerous tiny hypoechoic nodulations or diffuse homogeneous hypoechogenicity with echogenic fibrous bands [1-4]. An abnormal thyroid gland pattern on US not only helps the diagnosis of asymptomatic DTD [16,27] but it can also be a good diagnostic predictor in patients with subclinical to overt hypothyroidism when combined with TPOAb and TgAb [28,29]. Furthermore, US findings of the thyroid gland can predict outcomes of levothyroxine treatment in patients with subclinical hypothyroidism [29].

Thyroid cancer is one of the most common cancers in the Korean population, and reported to be 64.4/100,000 [30]. Recently, the incidence of thyroid cancer has rapidly increased in Korea because the increasing use of high-resolution US and US-FNAs have enabled the detection of subclinical disease [30-32]. On the other hand, thyroid cancer is very rare in central Europe and comprises only a 3/100,000 incidence rate with high incidence of benign nodules [33]. Well acknowledged suspicious US features suggesting malignancy in thyroid nodules are microlobulated or irregular margins, microcalcifications, hypoechogenicity, and taller than wide shape [13]. Although US is a powerful modality for differentiating malignancy from benign focal thyroid nodules [13,15,34], some studies have speculated that it might be difficult to detect malignant nodules in patients with HT on US because the heterogeneous hypoechogenicity and micronodulation seen in HT are somewhat similar to features seen in malignant thyroid nodules [2,12]. In this study, we evaluated whether the underlying thyroid parenchyma echogenicity affects the analysis of a thyroid nodule and which associated US features impact the analysis of a thyroid nodule on US. This study reveals that the underlying heterogeneous echogenicity of the background thyroid gland influences differentiation between benign and malignant thyroid nodules on US. The diagnostic performance of US had a more superior specificity, PPV and accuracy for diagnosing malignant nodules when thyroid glands showed underlying homogeneous echogenicity rather than heterogeneous echogenicity. Because the underlying heterogeneous echogenicity of the thyroid gland did affect the diagnostic performance of US, we wanted to evaluate which associated US features influenced the analysis of thyroid nodules on US. Among the benign nodules, microlobulated or irregular margins were more frequently seen in thyroid nodules with underlying heterogeneous echogenicity of the thyroid gland. In other word, physicians may have a higher chance to interpret a benign nodule as microlobulated or irregular margin on US in the underlying heterogeneous thyroid echogenicity group than in the underlying homogeneous thyroid echogenicity group, which explains the lower specificity of US in the underlying heterogeneous thyroid echogenicity group.

The US feature of focal lymphocytic thyroditis is variable; they can present either as hyperechoic nodules with ill-defined margins, ill-defined hypoechoic nodules, or solid hypoechoic nodules with well-defined margins [6,11,14,35]. However, a majority of studies reveal that margins of such nodules are often irregular [6,35] which can mimic suspicious malignant nodules on US and consequently increase the false positive rate of US. Even after excluding the nodules which were diagnosed with lymphocytic thyroditis, microlobulated or irregular margins were still more frequently observed in benign thyroid nodules with underlying heterogeneous thyroid echogenicity than in those with underlying homogeneous thyroid echogenicity. Therefore, our result supports the hypothesis that the underlying heterogeneous echogenicity of the thyroid gland can influence the differentiation of benign and malignant nodules, especially the US analysis of margins of thyroid nodules, a conclusion which needs verification with further studies.

There are some limitations to this study. First, some of the lesions that had undergone US-FNA only once were also included and considered benign or malignant. Although we believe that false-negative and false-positive results were negligible in our institution [36], the results of our study may be affected. Second, seven radiologists with varied experience performed US examinations and US-FNA, and interobserver variability among the radiologists may exist [34,37]. Third, the underlying parenchymal echogenicity of the thyroid gland was only classified into two categories in this study – homogeneous echogenicity and heterogeneous hypoechogenicity – and subcategories were not considered. Furthermore, due to interobserver variability, these categories may depend and vary among US performers. Fourth, this study population only included thyroid nodules which had been performed US-FNA. In our institution, US-FNAs are usually performed either on the thyroid nodule with suspicious US features or on the largest nodule if there are no suspicious US features. In this study, US-FNA had been performed on only one nodule in most cases. Therefore, a selection bias may exist. However, this study focused on the impact on underlying thyroid echogenicity for diagnosing thyroid malignancies using US, not the effect on several US features for diagnosing thyroid malignancies according to the multiplicity. Therefore, we do not think that this limitation has a strong influence on the value or result of this study.

## Conclusions

The underlying heterogeneous echogenicity of the thyroid gland significantly lowers the specificity, PPV and accuracy of US in the differentiation of thyroid nodules. Therefore, caution is required during evaluation of thyroid nodules detected among thyroid parenchyma showing heterogeneous echogenicity on US.

## Abbreviations

DTD: Diffuse thyroid disease; HT: Hashimoto thyroiditis; US: Ultrasonography; PTC: Papillary thyroid carcinoma; US-FNA: US-guided fine needle aspiration; TPOAb: Thyroid peroxidase antibody; TgAb: Thyroglobulin antibody; TBII: TSH-binding inhibitory immunoglobulins; PPV: Positive predictive value; NPV: Negative predictive value.

## Competing interests

The authors declare that they have no competing interests.

## Authors’ contributions

MP was involved in acquisition of data, analysis and interpretation of data and manuscript construction. SHP was involved in acquisition of data and revision. E-KK was involved in manuscript drafting and revision. JHY participated in study design and manuscript revision. HJM was involved in manuscript drafting and revision. HSL was involved in analysis and interpretation of data and revision. JYK mainly contributed to conception and decision, drafting the manuscript and final approval of the version to be published. All authors read and approved the final manuscript.

## Pre-publication history

The pre-publication history for this paper can be accessed here:

http://www.biomedcentral.com/1471-2407/13/550/prepub
